# Feature Ranking and Screening for Class-Imbalanced Metabolomics Data Based on Rank Aggregation Coupled with Re-Balance

**DOI:** 10.3390/metabo11060389

**Published:** 2021-06-14

**Authors:** Guang-Hui Fu, Jia-Bao Wang, Min-Jie Zong, Lun-Zhao Yi

**Affiliations:** 1 School of Science, Kunming University of Science and Technology, Kunming 650500, China; wangjiabao@126.com (J.-B.W.); zongminjie@126.com (M.-J.Z.); 2 Faculty of Agriculture and Food, Kunming University of Science and Technology, Kunming 650500, China

**Keywords:** class-imbalance, feature screening, rank aggregation, re-balance, filtering algorithm

## Abstract

Feature screening is an important and challenging topic in current class-imbalance learning. Most of the existing feature screening algorithms in class-imbalance learning are based on filtering techniques. However, the variable rankings obtained by various filtering techniques are generally different, and this inconsistency among different variable ranking methods is usually ignored in practice. To address this problem, we propose a simple strategy called rank aggregation with re-balance (RAR) for finding key variables from class-imbalanced data. RAR fuses each rank to generate a synthetic rank that takes every ranking into account. The class-imbalanced data are modified via different re-sampling procedures, and RAR is performed in this balanced situation. Five class-imbalanced real datasets and their re-balanced ones are employed to test the RAR’s performance, and RAR is compared with several popular feature screening methods. The result shows that RAR is highly competitive and almost better than single filtering screening in terms of several assessing metrics. Performing re-balanced pretreatment is hugely effective in rank aggregation when the data are class-imbalanced.

## 1. Introduction

Datasets with imbalanced distribution are quite common in classification. In the settings of binary category, a dataset is called “imbalanced” if the number of one class is far larger than the others in the training data. Generally, the majority class is called negative while the minority class is called positive. Thus, the number of positive instances is often much lower than that of negative ones.

A hindrance in class-imbalance learning is that standard classifiers are often biased towards the majority classes. Therefore, there is a higher misclassification rate in the minority instances [1,2]. Re-sampling is the standard strategy to deal with class-imbalance learning tasks. Many studies [2,3,4] have shown that re-sampling the dataset is an effective way to enhance the overall performance of the classification for several types of classifiers. Re-sampling methods concentrate on modifying the training set to make it suitable for a standard classifier. There are generally three types of re-sampling strategies to balance the class distribution: over-sampling, under-sampling, and hybrid sampling.

- Over-sampling adds a set sampled from the minority class. Randomly duplicating the minority instances, SMOTE [5] and smoothed bootstrap [6] are three widely used over-sampling methods.
- Under-sampling removes some of the data points from the majority class to alleviate the harms of imbalanced distribution. Random under-sampling (RUS) is a simple but effective way to randomly remove part of the majority class.
- Hybrid-sampling is a combination of over-sampling and under-sampling.

Let *D* be a dataset with *p* features $x_{1},x_{2},\ldots ,x_{p}$, the target of feature screening is to extract a part of features $x_{1}^{{}^{\prime }},x_{2}^{{}^{\prime }},\ldots ,x_{m}^{{}^{\prime }}$ such that m<<p and these selected features satisfy the specified conditions of the task at hand [7]. For instance, the target is to select the subset of candidate features to maximize classifier accuracy in a classification setting. In the past two decades, many papers in studies have adopted the feature screening methods [8,9,10]. Feature screening has many advantages such as reducing susceptibility to over-fitting, training models faster and offsetting the pernicious effects of the curse of dimensionality [8]. The disadvantage of feature screening is that some crucial features may be omitted, thus harming classification performance.

Filtering [11], wrapping [12], and embedding [13] are three kinds of approaches for feature screening. Filter algorithms screen top-ranked variables via a certain metric. Wrapper methods perform a search in all the combinations to find the best subsets of all features. Generally, a complete search is often time-consuming and greedy, so the heuristic technique is frequently utilized to explore the solutions. Embedded algorithms screen important variables while building the classifier. Of all the three types of feature screening, filter methods are the simplest and the most frequently used to solve real-world imbalanced problems [14] in class-imbalance learning community. Many metrics have been utilized to perform filtering feature screening algorithms, such as *t* test, Fisher score [15], Hellinger distance [16], Relief [17], ReliefF [18], information gain [19], Gini index [20], AUCROC [21], AUCPRC [22], geometric mean [23], F-measure [24], and R-value [25].

Ensemble feature selection has been widely applied to the field of classification [26], such as Nazrul et al. [27] provided an ensemble feature selection method using feature–class and feature mutual information to select an optimal subset of features by combining multiple subsets of features. Yang et al. [28] proposed an ensemble-based wrapper approach for feature selection from data with highly imbalanced class distribution. Nowadays, feature selection methods are popular in metabolomics data analysis. In order to resolve the problem of filtering the discriminative metabolites from high-dimension metabolomics data, Lin et al. [29] proposed a mutual information (MI)-SVM-RFE method that filters out noise and non-informative variables by means of artificial variables and MI, then conducts SVM-RFE to select the most discriminative features. Fu et al. [30] proposed two feature selection algorithms that, by minimizing the overlap degree between the majority and the minority, are effective in recognizing key features and control false discoveries for class-imbalanced metabolomics data. The above feature screening methods are usually established for balanced datasets, but they are also directly utilized in class-imbalance situations.

Different filtered approaches give different feature rankings because of their different theories, even when just counting top-ranked features. Motivated by this problem, we propose a simple strategy called rank aggregation with re-balance (RAR) to combine all methods’ ranking results in this study. It is an essential tool to fuse each rank to generate a synthetic rank that takes every ranking into account for class-imbalanced data. Different from the general feature selection methods, the proposed method combines different feature selection methods rather than simply accepting the result of one method, which can enhance the stability of the algorithm. At the same time, the great performances of the experiments in balanced and imbalanced metabolomics datasets verify the strong generalization abilities of RAR.

## 2. Results

### 2.1. Kendall’s τ Rank Correlation of Eight Filtering Methods on Class-Imbalanced Data

Each filtered method above can be employed to perform feature screening. However, we noted that different filtering feature screening techniques may give different rankings, especially when the data are extremely class-imbalanced. In this section, we compare methods using Kendall’s τ rank correlation [31].

**Figure 1:**
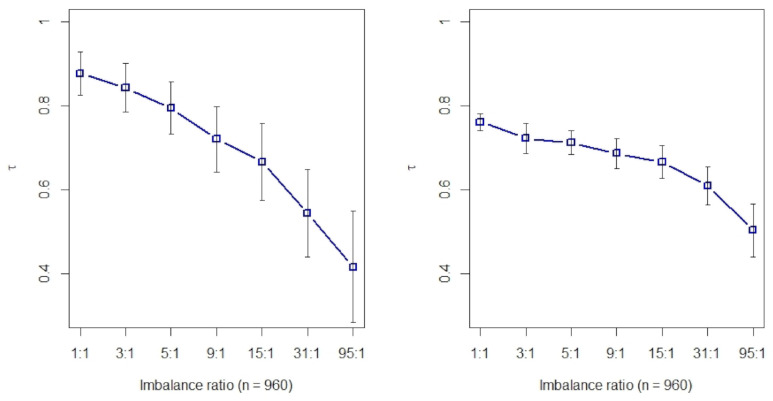
Kendall’s τ rank correlation coefficient under different imbalance ratios (with 100 repeats). **Left**: eight key variables; **right**: eight key plus eight irrelevant variables.

The Kendall’s τ rank correlation of eight filtering methods (*t* test, Fisher score, Hellinger distance, Relief, ReliefF, Information gain, Gini index, and R-value) are computed with simulated data that are generated by multivariate normal distributions, namely, $X|\left(y=0\right)∼N_{p}\left(\mu_{0},\Sigma \right)$ and $X|\left(y=1\right)∼N_{p}\left(\mu_{1},\Sigma \right)$, where the label y=0 denotes the majority class and y=1 minority class, respectively. The predictors in two classes have the same covariance matrix Σ, which is set to be a unit matrix for the purpose of simplicity. Two cases are considered in this study. In case one, the number of p=8, and eight variables are all set to be key features. The difference of mean values $\mu_{1}-\mu_{0}=\left[2.4,2.2,2,1.8,1.6,1.4,1.2,1\right]$. In case two, p=16, and the first eight variables are set to be the same with case one, but another eight irrelevant predictors are added. The number of total instances is set to 960. The negative to the positive ratios here are set be 1:1, 3:1, 9:1, 31:1, and 95:1, respectively. There are 28 Kendall’s τ rank correlation coefficients among 8 filtering methods, and the mean of these coefficients (with 100 repeats) is shown in Figure 1. As stated above, τ=1 if all pairs are concordant. Whereas the maximum of τ is 0.88 in case one (left, Figure 1) where there are no irrelevant predictors, and 0.76 in case two (right, Figure 1) where one-half of features are irrelevant variables. The two maximal τ values are reached when two classes are exactly balanced, and τ reduces as the imbalance ratio increases in two cases. It indicates that these filtering methods probably generate different feature rankings, and such differences tend to be intensified when the class imbalanced ratio increases. Consequently, it is hard to say that a filtering approach is better or worse than another one, and it is a big risk to just depend on a single filter algorithm to make decisions. We have known that such a difference will occur due to the different principles of the filtering methods, but we also presume that class imbalance intensifies this difference. A natural way to combat this challenge may combine each filtering approach’s information and relieve the effect of class imbalance. This is the motivation for why we propose the strategy of rank aggregation with re-balance.

### 2.2. Rank Aggregation(RA) on Original Balanced Data

**Figure 2:**
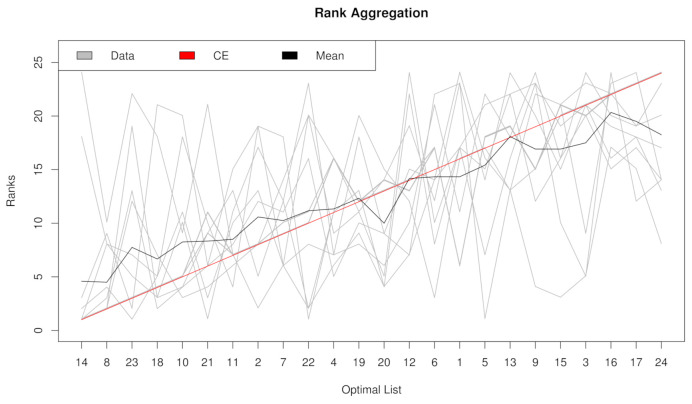
Rank lists of rank aggregation with the dataset “NPC” in case 1.

In our computation, eight filtering methods—*t* test, Fisher score, Hellinger distance, Relief, ReliefF, Information gain, Gini index, and R-value—are aggregated to generate an incorporative rank. Rank aggregation is firstly tested with the original balanced dataset “NPC”. Artificial rebalancing is unnecessary, and just case 1 (no resampling) is performed. Rank aggregation is compared with eight filtering methods: *t* test, Fisher score, Hellinger distance, Relief, ReliefF, Information gain, Gini index, and R-value. Gmean, $F_{1}$, AUCROC and AUCPRC are utilized as evaluation measurements. The rank lists ordered by their importance are shown on the *x*-axis in Figure 2. The top seven features are selected according to all four assessment metrics.

### 2.3. Rank Aggregation with Re-Balance (RAR) on Imbalanced Data

**Figure 3:**
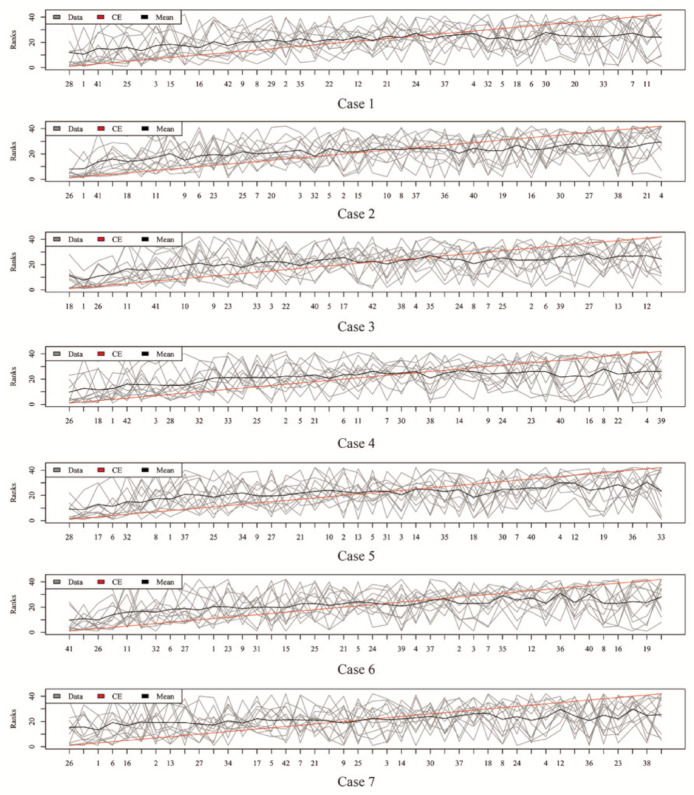
Rank lists of rank aggregation with the dataset “TBI” on seven cases.

**Figure 4:**
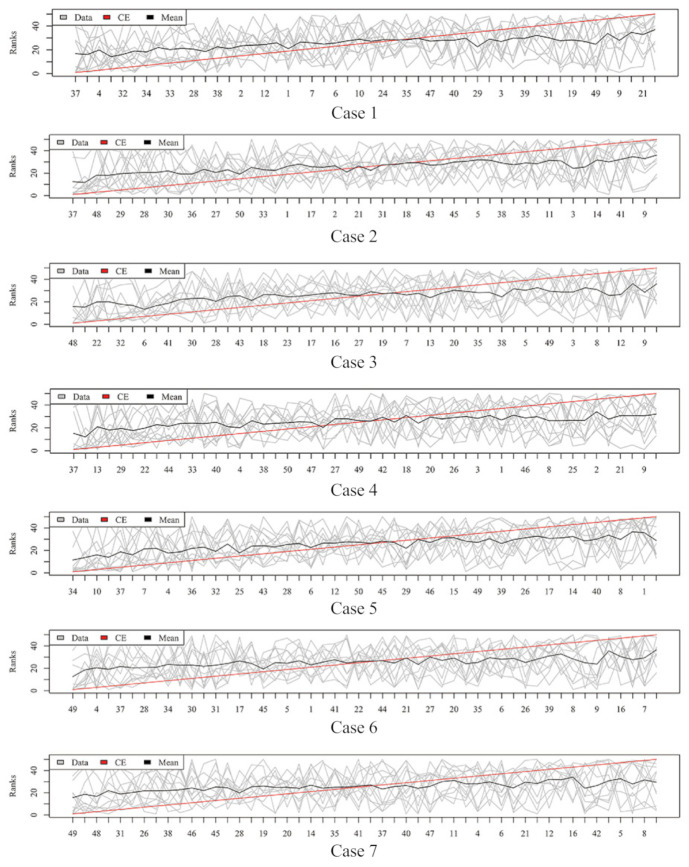
Rank lists of rank aggregation with the dataset “CHD2-1” on seven cases.

**Figure 5:**
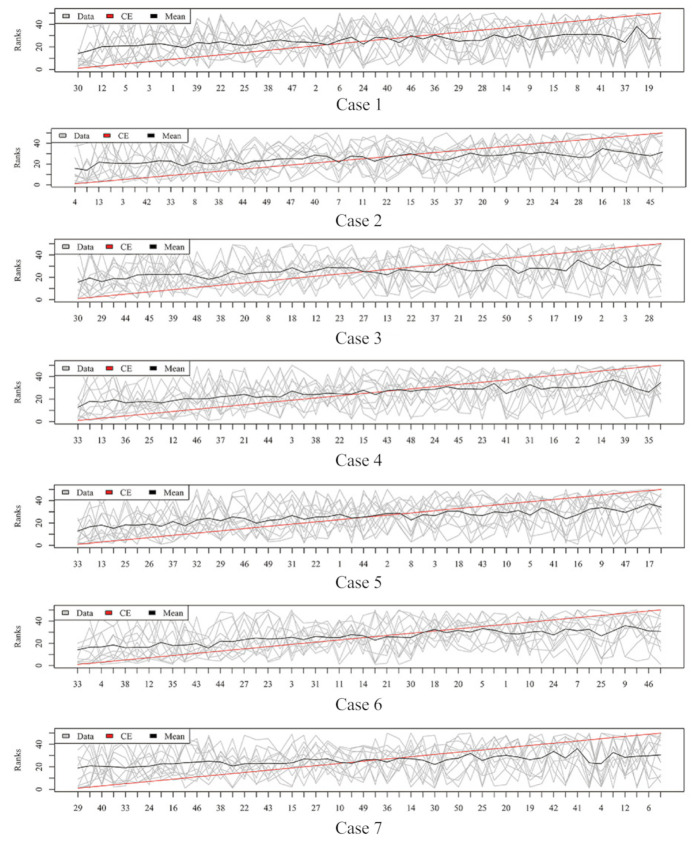
Rank lists of rank aggregation with the dataset “CHD2-2” on seven cases.

**Figure 6:**
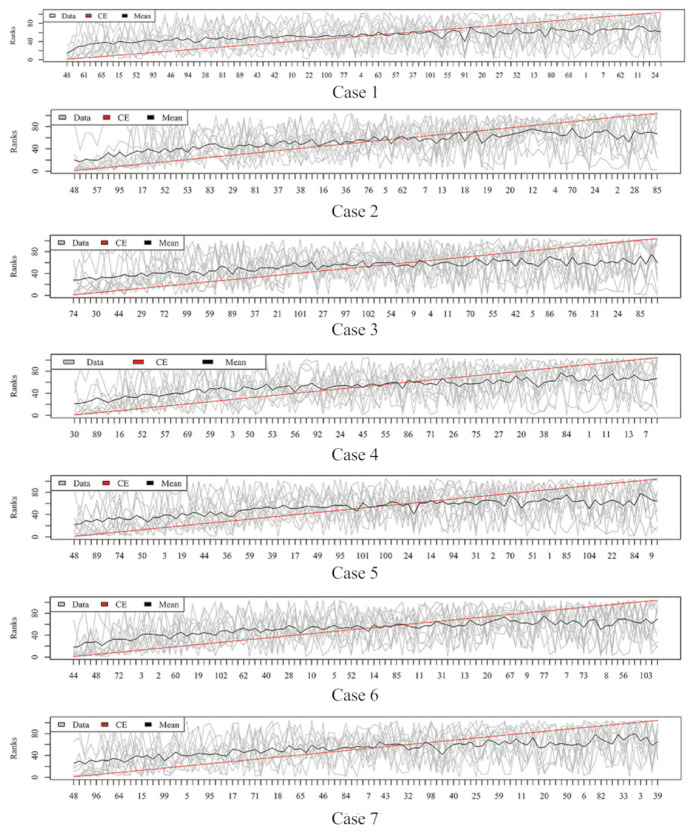
Rank lists of rank aggregation with the dataset “ATR” on seven cases.

Figure 3, Figure 4, Figure 5 and Figure 6 show the aggregated rank lists on seven cases with the datasets “TBI”, “CHD2-1”, “CHD2-2”, and “ATR”, respectively. Rank aggregation combines each ranking into a list reflective of the overall preference, and each subgraph of four figures shows the aggregation results based on the CE algorithm. The *x*-axis is the optimal list obtained by the rank aggregation algorithm. The *y*-axis also ranks, and the gray line is the rank of the original data; the black line is their average rank; and the red line is the aggregate result of the CE algorithm. The order of the *x*-axis rank is based on the aggregate ranks obtained by the red line. The performances measured by Gmean, $F_{1}$, AUCROC, and AUCPRC are given in Table 1, Table 2, Table 3 and Table 4, respectively.

## 3. Discussion

Table 1 and Table 2 show that RA reached the maximal values of Gmean and $F_{1}$. It can be seen from Table 3 and Table 4 that RA and ReliefF obtained the maximal values of AUCROC and AUCPRC. Therefore, RA outperformed single filtering methods when assessed with Gmean, $F_{1}$, AUCROC, and AUCPRC. The NPC dataset had a completely balanced distribution, and RA worked well on it. Thus, rank aggregation is necessary to integrate different results, even if in a totally balanced situation, and a consensual feature ranking list is provided.

**Figure 7:**
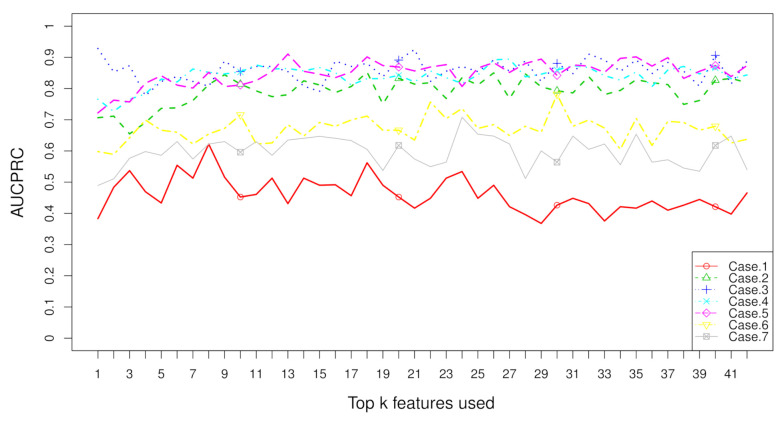
AUCPRC when top k features are used with the dataset “TBI” on seven cases.

**Figure 8:**
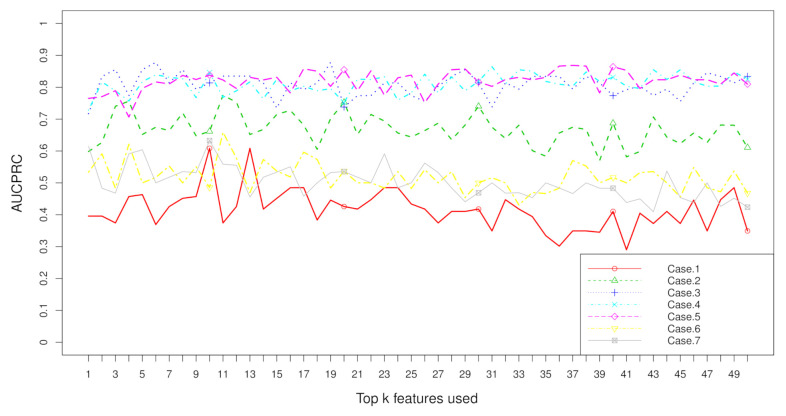
AUCPRC when top k features are used with the dataset “CHD2-1” on seven cases.

**Figure 9:**
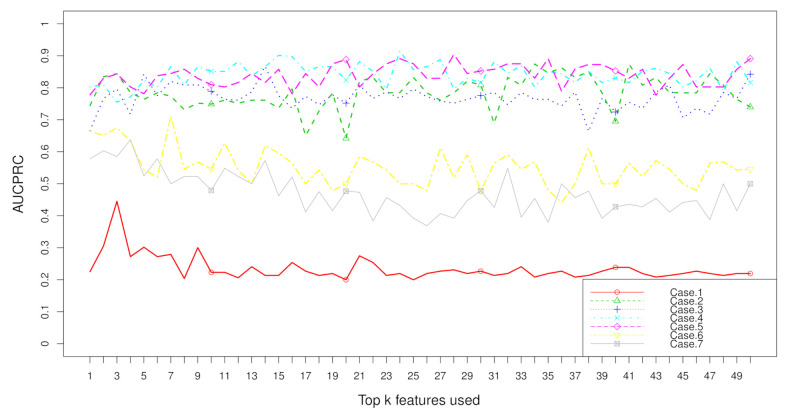
AUCPRC when top k features are used with the dataset “CHD2-2” on seven cases.

**Figure 10:**
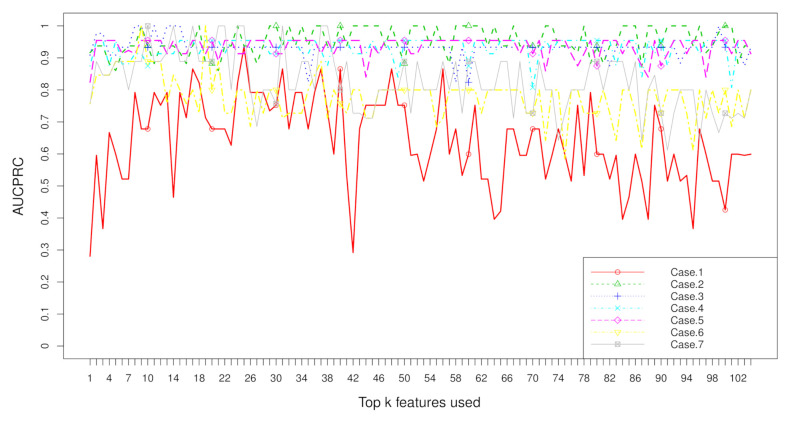
AUCPRC when top k features are used with the dataset “ATR” on seven cases.

Aggregation ranking lists in Figure 3, Figure 4, Figure 5 and Figure 6 tell us the order of importance of each feature. Though the rank lists derived from different subsampling methods were not the same, the top features were approximately consistent. After obtaining the rank list, another task is to figure out how many features should be considered as key variables. In this computation, we performed 5-fold cross-validation [32] to find the optimal number of key features. As recent studies have showed that AUCPRC is more informative in imbalanced learning [32,33], AUCPRC was employed as perfomance metric in this section, and random forest classifier was utilized to implement classification. Namely, the value of AUCPRC was calculated, as the top *k* ranked features were used each time, where *k* varies from 1 to *p* (see Figure 7, Figure 8, Figure 9 and Figure 10). We chose the optimal *k*- value such that the random forest classifier had the maximal AUCPRC in identifying classification. It can be seen from Table 1, Table 2, Table 3 and Table 4 that the optimal number of important features varied greatly under different re-balanced strategies. One possible reason is that the artificial data generated by different subsampling have difference to some extent. Another possible reason is the measurement changes sightly as the number of candidate features changes. This seems to be true from the Figure 7, Figure 8, Figure 9 and Figure 10 where each curve tends to be flat as the changes in the number of features used for classification. It also noted that the AUCPRC under no re-sampling (case 1) was generally lower than that under six re-sampling methods.

Table 1, Table 2, Table 3 and Table 4 report the results of these real datasets with the assessing metrics Gmean, $F_{1}$, AUCROC, and AUCPRC, respectively. We can perform comparisons in several aspects. Original imbalanced datasets are employed in case 1 from Table 1, Table 2, Table 3 and Table 4 (except NPC dataset). Of all the 16 “no re-balance” situations, the aggregation rank method reached the maximal measures in 12 situations compared with the other 8 filtering methods (t test, Fisher score, Hellinger distance, Relief, ReliefF, information gain, Gini, and R-value). It indicates that aggregation rank was better than a single filtering rank with the proportion of 75.00% when the data are class-imbalanced. If the original dataset NPC was counted in, this proportion was 80.00%. Therefore, rank aggregation is generally superior to single filtering methods, no matter how the data are balanced or imbalanced.

Re-balanced datasets were artificially generated and utilized in cases 2–7 from Table 1, Table 2, Table 3 and Table 4. Of all the 96 scenarios with re-balance, the aggregation rank method reached the maximal measures in 83 scenarios compared to the other eight filtering methods. It means that aggregation rank outperformed single filtering rank with the proportion of 86.46% when the class-imbalanced data were treated with re-balance strategies. Thus, performing aggregation rank is extremely effective in dealing with class-imbalanced data.

Rank aggregation was performed on both imbalanced datasets (RA) and re-balanced datasets (RAR). Of all the 96 scenarios with re-balance (cases 2–7 in Table 1, Table 2, Table 3 and Table 4), there were 93 situations whose measurements were equal or greater than those from case 1 (no re-balance). It shows that aggregation rank with re-balance strategies performed better with the proportion of 96.88% than that with original class-imbalanced data. Therefore, performing re-balance can play a crucial role in improving the performance of rank aggregation when the data are class-imbalanced.

Figure 7, Figure 8, Figure 9 and Figure 10 show the AUCPRC curves of seven cases on four imbalanced datasets. AUCPRC from re-balanced data (cases 2–7) was generally higher than that from imbalanced data (case 1). In other words, the performance can be promoted after re-sampling to balance the imbalanced data artificially. Case 5 and case 6 are two under-sampling methods, and the AUCPRC was generally lower than that from over-sampling or hybrid sampling (cases 2–4). The possible reason is that some of the useful information is missed in doing under-sampling when the size of the minority instances is too small (see Table 5). Therefore, one should be cautious about using under-sampling in practice.

In sum, different filter methods generate different rankings. Rank aggregation is necessary to integrate different results and provide a consensual feature ranking list. Class-imbalance usually leads to degraded performance from a filtering method on feature importance ranking. This harmfulness can be alleviated via different re-balance strategies in sample space.

## 4. Materials and Methods

### 4.1. Notations

The notations used in this study are listed below:


*D*
a dataset with two classes $C_{1}$ and $C_{2}$
$C_{1}$
the minority (positive) class
$C_{2}$
the majority (negative) class
*n*
the size of the total instances in *D*
*p*
the number of the features in *D*
$n_{k}$
the size of $C_{k}$, k=1,2
|D|
the number of samples in *D*
$x_{j}$
the *j*th feature, j=1,2,…,p
$x_{i}$
the *i*th instance, i=1,2,…,n
$\mu_{kj}$
the expectation of *j*th feature in $C_{k}$, k=1,2; j=1,2,…,p
$\sigma_{kj}^{2}$
the variance of *j*th feature in $C_{k}$, k=1,2; j=1,2,…,p
${\bar{x}}_{kj}$
the sample mean of *j*th feature in $C_{k}$, k=1,2; j=1,2,…,p
$s_{kj}^{2}$
the sample variance of *j*th feature in $C_{k}$, k=1,2; j=1,2,…,p

### 4.2. Eight Filtering Methods

#### 4.2.1. *t* Test

Feature screening using the *t* test statistic [34] is similar to performing a hypothesis test (the null hypothesis is that there is no difference in the means) on the class’s distribution, and its significance indicates the difference between majority and minority classes. The lower the *p* value of this *t* test, the higher the majority and minority classes’ significant difference. Consequently, the considered feature is more relevant to the separation of two classes.

#### 4.2.2. Fisher Score

Fisher score [35] is simple and generally quite effective, which can be a criterion of feature screening. Fisher score of a single feature is defined as follows:

(1) $$Fisher\left(x_{j}\right)=\frac{\left|\mu_{1j}-\mu_{2j}\right|}{\sigma_{1j}^{2}+\sigma_{2j}^{2}},\;j=1,2,\ldots ,p,$$

$\mu_{1j}$, $\mu_{2j}$, $\sigma_{2j}^{2}$, and $\sigma_{2j}^{2}$ can be replaced by their corresponding sample statistics in computation, namely,

(2) $$Fisher\left(x_{j}\right)=\frac{\left|{\bar{x}}_{1j}-{\bar{x}}_{2j}\right|}{s_{1j}^{2}+s_{2j}^{2}},\;j=1,2,\ldots ,p$$

A feature with a large Fisher score is more crucial for discriminating the two categories.

#### 4.2.3. Hellinger Distance

Hellinger distance can be used to measure a distributional divergence [36]. Denoted the two normal distributions by *P* and *Q*, Hellinger distance is calculated as follows:

(3) $$D_{H}^{2}\left(P,Q\right)=2-2\sqrt{\frac{2\sigma_{1}\sigma_{2}}{\sigma_{1}^{2}+\sigma_{2}^{2}}}exp\left\{-\frac{{\left(\mu_{1}-\mu_{2}\right)}^{2}}{4(\sigma_{1}^{2}+\sigma_{2}^{2})}\right\},$$

where $\mu_{1}$, $\sigma_{1}^{2}$, $\mu_{2}$, and $\sigma_{2}^{2}$ are the expectation and variance of *P* and *Q*, respectively, and their corresponding sample statistics are used in practice [37]. The larger the Hellinger distance is, the more divergent the two distributions are.

#### 4.2.4. Relief and ReliefF

The Relief is an iteration method that tries to give each feature a score to indicate its level of relevance to the response [37,38]. Let $x_{i}$ be an instance; ${nearhit}_{i}$ and ${nearmiss}_{i}$ be its two nearest neighbors from the same class and the other class by the Euclidean distance, respectively. The score vector $s={\left(s_{1},s_{2},\ldots ,s_{p}\right)}^{T}$ is refreshed as follows:

(4) $$s_{j}⟵s_{j}-{\left(x_{ij}-nearhit_{ij}\right)}^{2}+{\left(x_{ij}-nearmiss_{ij}\right)}^{2},\;j=1,2,\ldots ,p,$$

where $x_{ij}$, $nearhit_{ij}$ and $nearmiss_{ij}$ are the *j*th element of $x_{i}$, ${nearhit}_{i}$ and ${nearmiss}_{i}$, respectively. A feature with a higher score is more crucial to the response. Though ReliefF [39] is originally developed for dealing with multi-class and noise datasets, it can be applied to binary classification cases. Compared with Relief that searches one nearest instance from the same class and one from the other class in updating the weights, ReliefF finds *k* nearest neighbors. Similarly, a feature with a higher score is more important to the response.

#### 4.2.5. Information Gain (IG)

Information gain [40] is the measurement of informational theory and can be utilized to assess the importance of a given feature. In the settings of binary classification, the information entropy of the set *D* is defined as follows:

(5) $$Ent\left(D\right)=-\sum_{k=1}^{2}\frac{n_{k}}{n}log_{2}\frac{n_{k}}{n}$$

Assuming that a discrete feature (attribute) x has *V* different values $\left\{x_{1},x_{2},\ldots ,x_{V}\right\}$, and $D_{v}$ is the subset of instance set *D* satisfying $x=x_{v}$. The information gain of the variable x is

(6) $$IG\left(D,x\right)=Ent\left(D\right)-\sum_{v=1}^{V}\frac{|D_{v}|}{n}Ent\left(D_{v}\right),$$

The larger the information gain is, the more important the feature is for separating the classes. A continuous feature should be discretized before using the IG metric.

#### 4.2.6. Gini Index

Gini index [41] fits binary digits, continuous numerical values, ordinal numbers, etc. It is a non-purity split method. Gini index of *D* is defined as follows:

(7) $$Gini\left(D\right)=1-\sum_{k=1}^{2}p_{k}^{2},$$

where $p_{k}$ is the probability that any instance belongs to $C_{k}$, and it is replaced with $n_{k}/n$, (k=1,2) in practice. If we divide *D* into *M* subsets $D_{1},D_{2},\ldots ,D_{M}$, the Gini index after splitting is:

(8) $$Gini_{split}\left(D\right)=\sum_{m=1}^{M}\frac{|D_{m}|}{n}Gini\left(D_{m}\right)$$

The smaller the Gini index is, the more important the feature is.

#### 4.2.7. R-Value

R-value [30,42] indicates the degree of overlap for the class-imbalanced dataset. R-value for a dataset *D* is defined as follows:

(9) $$R\left(D\right)=\frac{1}{n}\sum_{k=1}^{2}\sum_{m=1}^{\left|C_{k}\right|}\lambda (\left|kNN\left(P_{km},D-C_{k}\right)\right|-\theta ),$$

where

(10) $$\lambda \left(x\right)=\left\{\begin{array}{c} 1,\;if\;x>0,\\ 0,\;otherwise \end{array}\right.$$

where $kNN(P,C_{i})$ is the subset of *k* nearest neighbors of instance *P* that belong to the set of instances $C_{i}$, and θ is the threshold generally set to be k/2 [43]. The smaller the R-value is, the more important the feature is for discriminating the categories.

### 4.3. Four Evaluation Metrics

#### 4.3.1. Geometric Mean and F-Measure

True positive, true negative, false positive, and false negative are denoted by TP, TN, FP, and FN, respectively. Some common metrics are listed below:

$$\begin{array}{cc} & TPR=recall=\frac{TP}{TP+FN};\\ & TNR=\frac{TN}{TN+FP};\\ & FPR=\frac{FP}{FP+TN};\\ & precision=\frac{TP}{TP+FP};\\ & Gmean=\sqrt{TPR\times TNR};\\ & F_{1}=\frac{2precision\times recall}{precision+recall} \end{array}$$

The range of both Gmean and $F_{1}$ is [0,1]. The larger they are, the better the classifer works.

#### 4.3.2. AUCROC and AUCPRC

AUCROC is the area under the receiver operating characteristic curve (ROC) [44]. AUCPRC is the area under the precision recall curve (PRC) [45]. Both AUCROC and AUCPRC range from 0 to 1, and the larger they are, the better the classifier is built for the imbalanced learning. More details on AUCROC or AUCPRC can be found in our previous studies [37,46].

Gmean, $F_{1}$, AUCROC, and AUCPRC are more widely used than the metric Accuracy in class-imbalance learning. These metrics actually pay more attention to the minority samples.

### 4.4. Kendall’S τ Rank Correlation

Kendall’s τ rank correlation statistic [47] can be applied to calculate the degree of comparability between the feature rankings of two filtering techniques. Let the two feature rankings generated by two filters be

$$\begin{array}{cc} & f_{1}:\;r_{11},r_{21},r_{31},\ldots ,r_{p1},\\ & f_{2}:\;r_{12},r_{22},r_{32},\ldots ,r_{p2}, \end{array}$$

and there are no ties in each of ranking list. Then Kendall’s τ is calculated as follows,

(11) $$\tau \left(f_{1},f_{2}\right)=\frac{\sum_{i<j}sgn\left(r_{i1}-r_{j1}\right)sgn\left(r_{i2}-r_{j2}\right)}{p(p-1)/2},$$

where sgn(x) is the sign function, namely it equals 1 if *x* is positive and −1 if *x* negative. A pair of (i,j) is called concordant if $r_{i1}>r_{j1}$ and $r_{i2}>r_{j2}$ or $r_{i1}<r_{j1}$ and $r_{i2}<r_{j2}$. Otherwise, they are considered discordant. The numerator $\sum_{i<j}sgn\left(r_{i1}-r_{j1}\right)sgn\left(r_{i2}-r_{j2}\right)$ is the difference between the number of concordant pairs and the number of discordant pairs, and the denominator p(p−1)/2 is the number of all distinct pairs of *p* elements. The range of τ is [−1,1]. If τ=0, the correlation of two rankings is weak; if τ=−1, then all pairs will be discordant, and the two rankings are exactly opposite; if τ=1, then all pairs are exactly concordant [48].

### 4.5. Rank Aggregation with Re-Balance for Class-Imbalanced Data

**Figure 11:**
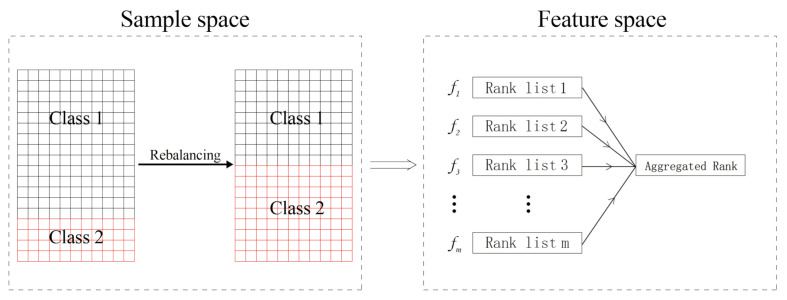
The frame of rank aggregation with re-balance.

As mentioned above, there are differences among the ranks from different filtering methods, but we assume that they are equal in match, namely, no one is better or worse than another. Rank aggregation (RA) is a greatly intuitive metric that computes the absolute differences between the ranks of all individual features [49]. Rank aggregation with re-balance (RAR) consists of two stages for class-imbalanced data and is illustrated in Figure 11. In sample space, the data are artificially balanced by generating new instances of the minority class or (and) removing some of the majority class instances. In feature space, *m* rank lists are first computed using *m* different filtering methods. Each rank list is the full permutation of all the features. Then, they are merged to be an aggregated rank. Feature screening and classification can be performed according to this aggregated rank.

#### 4.5.1. Rank Aggregation

As mentioned above, different filter techniques will give different feature ranking results. The rank aggregation method [34,50] combines all the rankings together, by aggregating all feature ranking lists generated from different filtering methods.

RA is to find an optimal ranking $\delta^{*}$ such that

(12) $$\delta^{*}=\arg\min\sum_{i=1}^{K}w_{i}d\left(\delta ,f_{i}\right),$$

where $f_{i}$ is the *i*th feature ranking list, δ represents a ranking list with the same length of $f_{i}$, *d* is a distance function, and $w_{i}$ is the important weight related with list $f_{i}$. In this study, *d* is chosen to be the Spearman’s foot rule distance [50]:

(13) $$d\left(\delta ,L_{M}\right)=\sum_{t\in L_{M}\cup \delta }|M(r^{\delta }\left(t\right))-M\left(r^{L_{M}}\left(t\right)\right)\left|\times \right|r^{\delta }\left(t\right)-r^{L_{M}}\left(t\right)|$$

$L_{M}=\left\{A_{1}^{M},...,A_{m}^{M}\right\}$ denotes an ordered list of top *m* algorithms produced by the validation measure *M*. Let M(1),...,M(m) be the scores for the top *m* algorithms in $L_{M}$, where M(1) is the best score given by measure *M* and so on. Let $r^{M}\left(A\right)$ be the rank of *A* under *M* (1 means “best”) if *A* is within top *m*, and be equal to m+1; otherwise, $r^{\delta }\left(A\right)$ is defined likewise.

The optimization of the objective (12) is achieved by using the Monte Carlo cross-entropy (CE) algorithm [51,52]. CE Monte Carlo algorithm is a stochastic search method, which produces a “better” sample in the future, which is concentrated around an *x* that corresponds to an optimal $\delta^{*}$ [50].

#### 4.5.2. Strategies to Generate New Samples

Before performing rank aggregation, the training instances are to be modified to produce a more balanced class distribution. To achieve this task, new minority or (and) majority class samples need to be generated or drawn from the original dataset. We employ the following three strategies to gain new samples:

##### Randomly Sampling

In the over-sampling, some (all) the minority class instances are randomly duplicated; in the under-sampling, a portion of majority samples are randomly removed.

##### SMOTE

**Figure 12:**
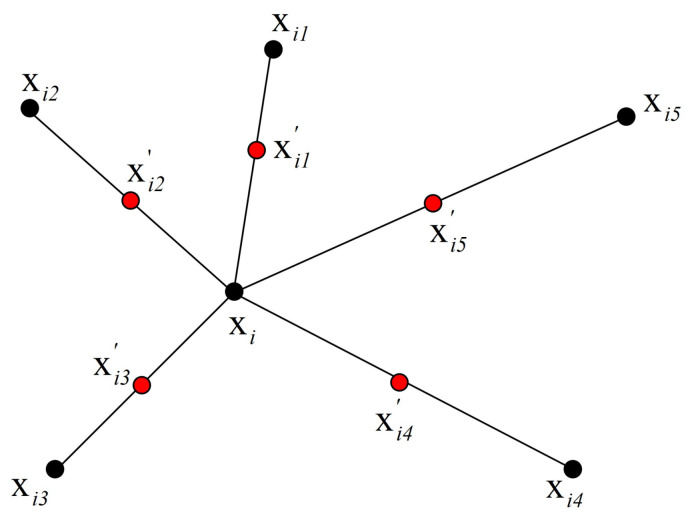
An illustration of how to create the synthetic data points in the SMOTE algorithm.

Synthetic minority over-sampling technique (SMOTE) is a popular over-sampling algorithm [5]. Figure 12 illustrates how to generate new samples according to the selected point $x_{i}$ in SMOTE. The five selected nearest neighbors of $x_{i}$ are $x_{i1}$ to $x_{i5}$. $x_{i1}^{{}^{\prime }}$ to $x_{i5}^{{}^{\prime }}$ are the synthetic data points created by the randomized interpolation. Namely,

$$x_{ih}^{{}^{\prime }}=x_{i}+u_{h}\left(x_{ih}-x_{i}\right),\;h=1,2,\ldots ,5,$$

where $u_{h}$ is a random number between 0 and 1. The above operation can be repeated to obtain requested synthetic minority instances.

##### Smoothed Bootstrap

Smoothed bootstrap technique repeatedly bootstraps the data from the two classes and employs smoothed kernel functions to generate new approximately balanced samples [53]. A new instance is generated by performing the following three steps:

step 1: choose y=k∈{1,2} with probability 0.5;
step 2: choose $\left(x_{i},y_{i}\right)$ in the original daa set such that $y_{i}=k$ with probability $1/n_{k}$;
step 3: sample x from a probability distribution $K_{H_{k}}\left(\cdot ,x_{i}\right)$, which is centered at $x_{i}$ and depends on the smoothing matrix $H_{k}$.

In brief, smoothed bootstrap firstly draws randomly from the original dataset an instance from one of the two categories, then generates a new instance in its neighborhood.

### 4.6. Experiment and Assessing Metrics

As shown in Table 5, five metabolomics datasets were employed to test our algorithm. NPC is a nasopharyngeal carcinoma dataset [32,54] that is exactly balanced. In this study, NPC was utilized to investigate the performance of rank aggregation strategy on original balanced data, which included 100 patients with nasopharyngeal carcinoma and 100 healthy controls. Traumatic brain injury (TBI) is from our previous studies [32,55], which reports the serum metabolic profiling of TBI patients with (or without) cognitive impairment (CI). The TBI dataset included 73 TBI patients with CI and 31 TBI patients without CI. CHD2-1 and CHD2-2 datasets are actually from the same experiment about coronary heart disease (CHD) [30]. The CHD2-1 dataset contains 21 patients with CHD, and the CHD2-2 dataset contains 16 patients with coronary heart disease associated with type 2 diabetes mellitus (CHD-T2DM), which are compared with a control group of 51 healthy adults. ATR is an Acori Tatarinowii Rhizoma dataset, which included 21 samples collected from Sichuan Province, and 8 samples were from Anhui Province in China [56]. Table 5 lists the summary of five datasets; included are the numbers of attributes, total instances, the majority, the minority instances, and the imbalance ratio. The NPC dataset was utilized to test the performance of rank aggregation under original balanced distribution. The other four imbalanced data sets were used to evaluate the RAR algorithm with artificially re-balanced data.

This section shows the efficacy of the proposed RAR algorithm on one original balanced dataset and four class-imbalanced datasets and compares it with other filtering feature screening methods via several assessing metrics. Rank aggregation was performed under the following seven situations:

- Case1: no re-sampling: the original datasets are directly utilized to perform rank aggregation. Denoted case 1 by “RA” because there is no re-sampling in it.
- Case2: hybrid-sampling A: some instances of the majority class are randomly eliminated, and new synthetic minority examples are generated by SMOTE. The size of the remaining majority is equal to the size of the (original plus new generated) minority class.
- Case3: hybrid-sampling B: A new synthetic dataset is generated according to the smoothed bootstrap re-sampling technique. The sizes of the majority and minority classes are approximately equal.
- Case4: over-sampling A: new minority class instances are randomly duplicated according to the original minority group.
- Case5: over-sampling B: new synthetic minority examples are generated on the basis of the smoothed bootstrap re-sampling technique.
- Case6: under-sampling A: some instances from the majority class are randomly removed so that the size of the remaining majority class is equal to the size of the minority.
- Case7: under-sampling B: new synthetic majority examples are generated according to the smoothed bootstrap re-sampling technique.

Note that NPC is balanced, and just case 1 is performed on it. Table 6 lists the summary of the six re-balanced strategies. In this study, Gmean, $F_{1}$, AUCROC, and AUCPRC are employed to assess the performance of RA or RAR algorithm on five datasets under seven cases.

## 5. Conclusions

In this paper, we propose a simple but effective strategy called RAR for feature screening of class-imbalanced data by aggregating rankings from individual filtering algorithms and modifying the class-imbalanced data with various re-sampling methods to provide balanced or more adequate data. RAR can address the problem of inconsistency between different feature ranking methods to a large extent. The results on real datasets show that RAR is highly competitive and almost better than single filtering screening in terms of geometric mean, F-measure, AUCROC, and AUCPRC. After performing re-balanced pretreatment, the performance of rank aggregation can be highly improved, so re-sampling to balance the classes is extremely useful in rank aggregation when the data are class-imbalanced in metabolomics. Our proposed method serves as a reference for future research on feature selection for the diagnosis of diseases.

Rank aggregation is a general idea to investigate the importance of features. In this study, rankings from eight filtering algorithms are employed to generate the aggregated rank. There are many other filter techniques, such as Chi-squared, power, Kolmogorov–Smirnov statistic, and signal-to-noise ratio [57], which are all widely utilized in class-imbalance learning. In addition, considering that a re-sampling method can also generate a rank list, rank aggregation can be performed according to the various re-sampling algorithms rather than different filtering methods. Further, if necessary, ensemble multiple rank aggregations could be performed to combine those aggregated rankings derived from different algorithms. Finally, although RAR is used in the metabolomics datasets in this study, it is potentially available for handing high-dimensional imbalanced data from other fields, such as economics and biology.

## Figures and Tables

**Table 1:** Gmean from rank aggregation and eight filtering techniques (The best result is in bold).

Dataset	Resampling	No.	RA	RAR	*t* Test	Fisher	Hellinger	Relief	ReliefF	IG	Gini	R-Value
NPC	Case 1	7	**1.00**	−	0.87	0.97	0.95	0.95	0.95	0.95	**1.00**	0.92
TBI	Case 1	6	**0.96**	−	0.41	0.68	0.68	0.70	0.88	0.72	0.58	0.63
	Case 2	11	−	**1.00**	0.84	0.88	0.95	0.90	0.94	0.86	0.90	0.90
	Case 3	1	−	**1.00**	0.95	0.84	0.95	0.90	0.80	0.95	0.90	**1.00**
	Case 4	10	−	**1.00**	0.96	**1.00**	0.89	0.93	0.97	0.93	0.85	**1.00**
	Case 5	7	−	**1.00**	0.93	0.90	0.97	0.97	0.93	0.97	0.86	0.93
	Case 6	12	−	**1.00**	0.75	0.83	0.82	0.83	0.91	0.85	0.71	**1.00**
	Case 7	29	−	**1.00**	0.71	0.70	0.65	0.82	0.85	0.78	0.71	0.71
CHD2-1	Case 1	10	**0.87**	−	0.67	0.71	**0.87**	0.00	0.77	0.77	0.47	**0.87**
	Case 2	11	−	0.94	0.85	0.85	**1.00**	0.87	0.93	0.94	0.85	0.91
	Case 3	6	−	**1.00**	0.86	**1.00**	**1.00**	**1.00**	0.93	**1.00**	0.93	**1.00**
	Case 4	31	−	**1.00**	**1.00**	**1.00**	**1.00**	**1.00**	**1.00**	**1.00**	0.95	**1.00**
	Case 5	37	−	**1.00**	**1.00**	**1.00**	**1.00**	**1.00**	**1.00**	**1.00**	**1.00**	**1.00**
	Case 6	11	−	0.87	0.61	0.75	0.87	0.87	**0.89**	0.87	0.50	0.87
	Case 7	10	−	**0.87**	0.61	0.71	**0.87**	**0.87**	**0.87**	**0.87**	0.71	0.71
CHD2-2	Case 1	3	0.58	−	0.48	0.58	0.48	0.58	0.55	0.68	0.00	**0.73**
	Case 2	34	−	**1.00**	**1.00**	**1.00**	**1.00**	**1.00**	**1.00**	**1.00**	**1.00**	**1.00**
	Case 3	14	−	**1.00**	**1.00**	**1.00**	**1.00**	**1.00**	**1.00**	**1.00**	**1.00**	**1.00**
	Case 4	24	−	**1.00**	**1.00**	**1.00**	**1.00**	**1.00**	**1.00**	**1.00**	**1.00**	**1.00**
	Case 5	28	−	**1.00**	**1.00**	**1.00**	**1.00**	**1.00**	**1.00**	**1.00**	**1.00**	**1.00**
	Case 6	7	−	**1.00**	**1.00**	0.82	0.82	**1.00**	0.82	0.82	0.47	0.82
	Case 7	4	−	0.86	0.82	**1.00**	0.82	**1.00**	0.61	**1.00**	0.00	0.75
ATR	Case 1	25	**1.00**	−	**1.00**	**1.00**	**1.00**	**1.00**	**1.00**	**1.00**	0.50	**1.00**
	Case 2	9	−	**1.00**	**1.00**	**1.00**	**1.00**	**1.00**	**1.00**	**1.00**	**1.00**	**1.00**
	Case 3	8	−	**1.00**	**1.00**	**1.00**	**1.00**	**1.00**	**1.00**	**1.00**	**1.00**	**1.00**
	Case 4	2	−	**1.00**	**1.00**	**1.00**	**1.00**	**1.00**	**1.00**	**1.00**	0.89	**1.00**
	Case 5	2	−	**1.00**	**1.00**	**1.00**	**1.00**	**1.00**	**1.00**	**1.00**	0.80	**1.00**
	Case 6	9	−	**1.00**	**1.00**	**1.00**	**1.00**	**1.00**	**1.00**	**1.00**	0.71	**1.00**
	Case 7	10	−	**1.00**	**1.00**	**1.00**	**1.00**	**1.00**	**1.00**	**1.00**	**1.00**	**1.00**

**Table 2:** $F_{1}$ from rank aggregation and eight filtering techniques (The best result is in bold).

Dataset	Resampling	NO.	RA	RAR	*t* Test	Fisher	Hellinger	Relief	ReliefF	IG	Gini	R-Value
NPC	Case 1	8	**1.00**	−	0.88	0.95	0.95	0.97	**1.00**	0.95	0.95	0.92
TBI	Case 1	11	**0.93**	−	0.82	0.86	0.87	0.90	0.90	0.88	0.83	0.88
	Case 2	13	−	**1.00**	0.84	0.90	0.94	0.95	0.94	0.84	0.87	0.86
	Case 3	1	−	**1.00**	0.87	0.87	**1.00**	**1.00**	0.96	0.95	0.83	**1.00**
	Case 4	7	−	**1.00**	0.91	0.97	0.93	0.97	0.90	0.93	0.89	**1.00**
	Case 5	10	−	**1.00**	0.87	0.93	0.93	0.93	0.93	0.97	0.88	0.93
	Case 6	19	−	**1.00**	0.73	0.80	0.86	0.86	0.77	0.80	0.71	0.91
	Case 7	11	−	**1.00**	0.83	0.92	0.80	0.86	0.86	0.75	0.50	0.86
CHD2-1	Case 1	10	**0.88**	−	0.87	**0.88**	0.87	0.87	0.87	0.87	0.83	0.87
	Case 2	11	−	**0.94**	**0.94**	0.71	0.93	0.89	0.93	0.88	0.67	0.89
	Case 3	6	−	**1.00**	0.89	**1.00**	**1.00**	**1.00**	0.93	0.94	0.94	0.92
	Case 4	31	−	**1.00**	0.95	0.95	0.96	0.90	0.96	0.90	0.86	0.95
	Case 5	37	−	**1.00**	**1.00**	0.91	0.95	0.95	**1.00**	0.95	0.86	0.95
	Case 6	11	−	0.89	0.50	0.75	0.73	0.89	**1.00**	0.83	0.55	0.67
	Case 7	10	−	**0.86**	0.73	0.57	0.80	0.75	**0.86**	0.67	0.55	0.57
CHD2-2	Case 1	3	0.91	−	0.87	0.86	**0.95**	0.87	0.91	0.90	0.87	0.91
	Case 2	34	−	**1.00**	**1.00**	**1.00**	0.88	0.92	0.93	**1.00**	0.88	0.93
	Case 3	14	−	**1.00**	**1.00**	0.92	0.86	0.92	**1.00**	0.93	0.86	0.93
	Case 4	24	−	**1.00**	0.90	0.91	0.95	0.95	0.95	**1.00**	0.95	0.95
	Case 5	28	−	**1.00**	0.95	0.95	0.95	0.95	**1.00**	0.96	0.95	**1.00**
	Case 6	7	−	**0.86**	0.57	0.80	0.80	0.75	**0.86**	**0.86**	0.50	**0.86**
	Case 7	4	−	**0.86**	0.80	0.75	0.57	**0.86**	**0.86**	**0.86**	0.50	0.67
ATR	Case 1	25	**1.00**	−	**1.00**	0.89	**1.00**	**1.00**	**1.00**	**1.00**	0.89	**1.00**
	Case 2	9	−	**1.00**	**1.00**	**1.00**	**1.00**	**1.00**	**1.00**	**1.00**	**1.00**	**1.00**
	Case 3	8	−	**1.00**	**1.00**	**1.00**	**1.00**	**1.00**	**1.00**	**1.00**	**1.00**	**1.00**
	Case 4	2	−	**1.00**	**1.00**	**1.00**	**1.00**	**1.00**	**1.00**	**1.00**	**0.89**	**1.00**
	Case 5	2	−	**1.00**	**1.00**	**1.00**	**1.00**	**1.00**	**1.00**	**1.00**	**1.00**	**1.00**
	Case 6	9	−	**1.00**	**1.00**	**1.00**	**1.00**	**1.00**	**1.00**	**1.00**	**0.67**	**1.00**
	Case 7	10	−	**1.00**	**1.00**	**1.00**	**1.00**	**1.00**	**1.00**	**1.00**	**0.67**	**1.00**

**Table 3:** AUCROC from rank aggregation and eight filtering techniques (The best result is in bold).

Dataset	Resampling	NO.	RA	RAR	*t* Test	Fisher	Hellinger	Relief	ReliefF	IG	Gini	R-Value
NPC	Case 1	16	**0.96**	−	0.90	0.91	0.94	0.93	0.96	0.93	0.93	0.95
TBI	Case 1	3	**0.70**	−	0.48	0.61	0.52	0.61	0.65	0.58	0.49	0.67
	Case 2	11	−	**0.89**	0.80	0.81	0.78	0.82	0.87	0.79	0.72	0.83
	Case 3	1	−	**0.95**	0.83	0.74	0.94	0.85	0.83	0.85	0.76	**0.95**
	Case 4	28	−	**0.91**	0.85	0.86	0.87	0.88	0.86	0.86	0.84	0.89
	Case 5	26	−	**0.93**	0.91	0.92	0.90	0.90	0.92	0.90	0.88	0.90
	Case 6	22	−	**0.77**	0.65	0.68	0.69	0.73	0.74	0.73	0.50	0.71
	Case 7	25	−	**0.71**	0.63	0.56	0.68	0.53	0.61	0.61	0.35	0.66
CHD2-1	Case 1	10	0.60	−	0.48	0.51	**0.61**	0.50	0.59	0.60	0.45	0.59
	Case 2	11	−	0.76	0.66	0.60	0.75	**0.77**	0.73	0.72	0.61	0.73
	Case 3	6	−	**0.92**	0.85	0.81	**0.92**	0.90	0.81	0.88	0.80	0.79
	Case 4	31	−	0.86	0.81	0.79	0.86	**0.87**	0.82	0.84	0.75	0.84
	Case 5	37	−	0.87	0.85	0.84	0.86	0.86	0.81	0.86	**0.90**	0.82
	Case 6	11	−	**0.64**	0.57	0.45	0.50	0.57	0.57	**0.64**	0.36	0.62
	Case 7	10	−	**0.60**	0.52	0.55	0.52	0.48	0.52	**0.60**	0.36	0.52
CHD2-2	Case 1	3	**0.60**	−	0.52	0.49	0.54	0.50	0.52	0.55	0.39	0.57
	Case 2	34	−	0.86	0.79	0.84	**0.88**	0.73	0.80	0.80	0.70	0.79
	Case 3	14	−	**0.90**	0.85	0.84	0.82	**0.90**	0.68	0.88	0.81	0.84
	Case 4	24	−	**0.93**	0.88	0.88	0.88	0.90	0.86	0.91	0.87	**0.93**
	Case 5	28	−	**0.91**	0.88	0.90	0.88	0.85	**0.91**	0.89	0.89	0.90
	Case 6	7	−	0.63	0.53	0.56	**0.66**	0.59	0.44	0.56	0.28	0.59
	Case 7	4	−	**0.66**	0.56	0.63	0.50	**0.66**	**0.66**	0.63	0.22	0.56
ATR	Case 1	25	0.88	**0.98**	−	0.51	0.85	0.85	0.76	0.85	0.50	0.76
	Case 2	9	−	**0.96**	0.79	0.86	**0.96**	**0.96**	0.93	**0.96**	0.75	0.93
	Case 3	8	−	0.97	**1.00**	0.90	0.97	0.90	0.97	0.97	0.80	0.93
	Case 4	2	−	**0.98**	0.93	0.83	0.88	**0.98**	0.95	**0.98**	0.81	0.95
	Case 5	2	−	**0.98**	0.81	0.86	0.76	0.93	0.93	0.95	0.71	0.88
	Case 6	9	−	**0.94**	0.81	0.75	0.88	**0.94**	0.88	0.81	0.19	0.81
	Case 7	10	−	**0.94**	0.81	0.63	0.69	0.63	**0.94**	0.88	0.19	0.75

**Table 4:** AUCPRC from rank aggregation and eight filtering techniques (The best result is in bold).

Dataset	Resampling	NO.	RA	RAR	*t* Test	Fisher	Hellinger	Relief	ReliefF	IG	Gini	R-Value
NPC	Case 1	15	**0.96**	−	0.87	0.90	0.91	0.92	0.96	0.92	0.91	0.91
TBI	Case 1	8	**0.62**	−	0.27	0.58	0.40	0.47	0.56	0.47	0.26	0.46
	Case 2	18	−	0.85	0.79	0.81	0.78	**0.86**	0.84	0.81	0.65	0.75
	Case 3	1	−	**0.93**	0.77	0.60	0.91	0.85	0.74	0.85	0.74	0.89
	Case 4	27	−	**0.89**	0.84	0.81	0.83	0.85	0.86	0.83	0.81	0.85
	Case 5	13	−	**0.91**	0.81	0.82	0.81	0.82	0.90	0.86	0.79	0.81
	Case 6	30	−	**0.78**	0.61	0.69	0.64	0.62	0.66	0.67	0.50	0.70
	Case 7	24	−	**0.71**	0.65	0.54	0.64	0.59	0.57	0.62	0.41	0.58
CHD2-1	Case 1	10	**0.61**	−	0.33	0.30	0.52	0.28	0.45	0.43	0.23	0.45
	Case 2	11	−	**0.77**	0.56	0.58	0.69	0.65	0.64	0.68	0.55	0.65
	Case 3	6	−	**0.88**	0.79	**0.88**	0.81	0.77	0.81	0.86	0.79	0.86
	Case 4	31	−	**0.86**	0.81	0.80	0.80	0.82	0.83	0.85	0.73	0.80
	Case 5	37	−	**0.87**	0.81	0.75	0.84	0.85	0.82	0.84	0.78	0.82
	Case 6	11	−	**0.66**	0.46	0.45	0.48	0.65	0.53	0.55	0.40	0.50
	Case 7	10	−	**0.63**	0.44	0.53	0.52	0.55	0.56	0.56	0.40	0.48
CHD2-2	Case 1	3	**0.45**	−	0.22	0.33	0.27	0.32	0.31	0.27	0.21	0.26
	Case 2	34	−	**0.87**	0.68	0.78	0.76	0.82	0.85	0.86	0.73	0.80
	Case 3	14	−	**0.87**	0.81	0.74	0.81	0.86	0.76	0.83	0.79	0.78
	Case 4	24	−	**0.91**	0.84	0.90	0.87	0.85	0.85	0.87	0.85	0.81
	Case 5	28	−	**0.90**	0.86	0.88	0.80	0.88	0.86	0.89	0.79	0.79
	Case 6	7	−	**0.71**	0.42	0.62	0.64	0.66	0.45	0.57	0.35	0.63
	Case 7	4	−	0.64	0.64	0.57	0.50	0.63	0.55	**0.66**	0.40	0.60
ATR	Case 1	25	**0.93**	−	0.75	0.52	0.52	0.82	0.82	0.82	0.25	0.60
	Case 2	9	−	**1.00**	0.81	0.88	0.92	0.94	0.94	0.94	0.68	0.88
	Case 3	8	−	**1.00**	**1.00**	0.78	**1.00**	0.88	0.93	**1.00**	0.82	0.88
	Case 4	2	−	**0.95**	0.84	0.79	0.91	0.91	0.88	0.91	0.70	0.91
	Case 5	2	−	**0.95**	0.79	0.78	0.78	0.88	0.90	**0.95**	0.61	0.88
	Case 6	9	−	**1.00**	0.89	0.69	0.85	0.89	0.76	0.85	0.36	0.80
	Case 7	10	−	**1.00**	0.61	0.54	0.64	0.54	0.80	0.89	0.37	0.80

**Table 5:** The summary of five datasets.

Datasets	Attributes	Instances	Majority	Minority	Ratio
NPC	24	200	100	100	1.00
TBI	42	104	73	31	2.35
CHD2-1	50	72	51	21	2.43
CHD2-2	50	67	51	16	3.19
ATR	104	29	21	8	2.63

**Table 6:** Re-balanced strategies.

Methods	Re-Sampling Process			Algorithm Process		
	**Under-Sampling**	**Over-Sampling**	**Hybrid**	**SMOTE**	**Random**	**Smoothed Bootstrap**
Case 2			Yes	Yes		
Case 3			Yes			Yes
Case 4		Yes			Yes	
Case 5		Yes				Yes
Case 6	Yes				Yes	
Case 7	Yes					Yes

## Data Availability

The datasets for this article are available from the corresponding author.

## References

[1] Brodley C., Friedl M. (1999). Identifying mislabeled training data. J. Artif. Intell. Res..

[2] Chawla N. (2009). Data mining for imbalanced datasets: An overview. Data Mining and Knowledge Discovery Handbook.

[3] Krawczyk B. (2016). Learning from imbalanced data: Open challenges and future directions. Prog. Artif. Intell..

[4] Cordón I., García S., Fernández A., Herrera F. (2018). Imbalance: Oversampling algorithms for imbalanced classification in R. Knowl. Based Syst..

[5] Chawla N., Bowyer K., Hall L., Kegelmeyer W. (2002). SMOTE: Synthetic minority over-sampling technique. J. Artif. Intell. Res..

[6] Lunardon N., Menardi G., Torelli N. (2014). ROSE: A Package for Binary Imbalanced Learning. R J..

[7] Hulse J.V., Khoshgoftaar T., Napolitano A., Wald R. Feature selection with high-dimensional imbalanced data. Proceedings of the 2009 IEEE International Conference on Data Mining Workshops.

[8] Guyon I., Elisseeff A. (2003). An introduction to variable and feature selection. J. Mach. Learn. Res..

[9] Saeys Y., Inza I., Larrañaga P. (2007). A review of feature selection techniques in bioinformatics. Bioinformatics.

[10] Yun Y.H., Li H.D., Deng B.C., Cao D.S. (2019). An overview of variable selection methods in multivariate analysis of near-infrared spectra. TrAC Trends Anal. Chem..

[11] Su X., Khoshgoftaar T. (2009). A survey of collaborative filtering techniques. Adv. Artif. Intell..

[12] Ambjørn J., Janik R., Kristjansen C. (2006). Wrapping interactions and a new source of corrections to the spin-chain/string duality. Nucl. Phys. B.

[13] Higman G., Neumann B., Neuman H. (1949). Embedding theorems for groups. J. Lond. Math. Soc..

[14] Guo H., Li Y., Shang J., Gu M., Huang Y., Gong B. (2017). Learning from class-imbalanced data: Review of methods and applications. Expert Syst. Appl..

[15] Gu Q., Li Z., Han J. (2012). Generalized fisher score for feature selection. arXiv.

[16] Yin L., Ge Y., Xiao K., Wang X., Quan X. (2013). Feature selection for high-dimensional imbalanced data. Neurocomputing.

[17] Spolaôr N., Cherman E., Monard M., Lee H. ReliefF for multi-label feature selection. Proceedings of the 2013 Brazilian Conference on Intelligent Systems.

[18] Kira K., Rendell L. (1992). The feature selection problem: Traditional methods and a new algorithm. Aaai.

[19] Lee C., Lee G. (2006). Information gain and divergence-based feature selection for machine learning-based text categorization. Inf. Process. Manag..

[20] Lerman R., Yitzhaki S. (1984). A note on the calculation and interpretation of the Gini index. Econ. Lett..

[21] Lobo J., Jiménez-Valverde A., Real R. (2008). AUC: A misleading measure of the performance of predictive distribution models. Glob. Ecol. Biogeogr..

[22] Boyd K., Eng K., Page C. (2013). Area under the precision-recall curve: Point estimates and confidence intervals. Joint European Conference on Machine Learning and Knowledge Discovery in Databases.

[23] Altidor W., Khoshgoftaar T., Napolitano A. Wrapper-based feature ranking for software engineering metrics. Proceedings of the 2009 International Conference on Machine Learning and Applications.

[24] Pillai I., Fumera G., Roli F. F-measure optimisation in multi-label classifiers. Proceedings of the 21st International Conference on Pattern Recognition (ICPR2012).

[25] Lee J., Batnyam N., Oh S. (2013). RFS: Efficient feature selection method based on R-value. Comput. Biol. Med..

[26] Ali M., Ali S.I., Kim D., Hur T., Bang J., Lee S., Kang B.H., Hussain M., Zhou F. (2018). UEFS: An efficient and comprehensive ensemble-based feature selection methodology to select informative features. PLoS ONE.

[27] Hoque N., Singh M., Bhattacharyya D.K. (2018). EFS-MI: An ensemble feature selection method for classification. Complex Intell. Syst..

[28] Yang P., Liu W., Zhou B.B., Chawla S., Zomaya A.Y. (2013). Ensemble-Based Wrapper Methods for Feature Selection and Class Imbalance Learning.

[29] Lin X., Yang F., Zhou L., Yin P., Kong H., Xing W., Lu X., Jia L., Wang Q., Xu G. (2012). A support vector machine-recursive feature elimination feature selection method based on artificial contrast variables and mutual information. J. Chromatogr. B.

[30] Fu G.H., Wu Y.J., Zong M.J., Yi L.Z. (2020). Feature selection and classification by minimizing overlap degree for class-imbalanced data in metabolomics. Chemom. Intell. Lab. Syst..

[31] Sen P. (1968). Estimates of the regression coefficient based on Kendall’s tau. J. Am. Stat. Assoc..

[32] Fu G.H., Xu F., Zhang B.Y., Yi L.Z. (2017). Stable variable selection of class-imbalanced data with precision-recall criterion. Chemom. Intell. Lab. Syst..

[33] Takaya S., Marc R., Guy B. (2015). The Precision-Recall Plot Is More Informative than the ROC Plot When Evaluating Binary Classifiers on Imbalanced Datasets. PLoS ONE.

[34] Yun Y.H., Deng B.C., Cao D.S., Wang W.T., Liang Y.Z. (2016). Variable importance analysis based on rank aggregation with applications in metabolomics for biomarker discovery. Anal. Chim. Acta.

[35] Weston J., Mukherjee S., Chapelle O. Feature selection for SVMs. Proceedings of the Advances in Neural information Processing Systems.

[36] Kailath T. (1967). The Divergence and Bhattacharyya Distance Measures in Signal Selection. IEEE Trans. Commun. Technol..

[37] Fu G.H., Wu Y.J., Zong M.J., Pan J. (2020). Hellinger distance-based stable sparse feature selection for high-dimensional class-imbalanced data. BMC Bioinform..

[38] Robnik-Šikonja M., Kononenko I. (2003). Theoretical and Empirical Analysis of ReliefF and RReliefF. Mach. Learn..

[39] Kononenko I. (1994). Estimating attributes: Analysis and extensions of RELIEF. European Conference on Machine Learning.

[40] Yang Y., Pedersen J. (1997). A comparative study on feature selection in text categorization. Icml.

[41] Shang W., Huang H., Zhu H., Lin Y., Qu Y., Wang Z. (2007). A novel feature selection algorithm for text categorization. Expert Syst. Appl..

[42] Borsos Z., Lemnaru C., Potolea R. (2018). Dealing with overlap and imbalance: A new metric and approach. Pattern Anal. Appl..

[43] Oh S. (2011). A new dataset evaluation method based on category overlap. Comput. Biol. Med..

[44] Provost F., Fawcett T. (2001). Robust classification for imprecise environments. Mach. Learn..

[45] Davis J., Goadrich M. The relationship between Precision-Recall and ROC curves. Proceedings of the 23rd International Conference on Machine Learning.

[46] Fu G.H., Yi L.Z., Pan J. (2019). Tuning model parameters in class-imbalanced learning with precision-recall curve. Biom. J..

[47] Kendall M.G. (1938). A New Measure of Rank Correlation. Biometrika.

[48] Shieh G. (1998). A weighted Kendall’s tau statistic. Stat. Probab. Lett..

[49] Pihur V. (2009). Statistical Methods for High-Dimensional Genomics Data Analysis.

[50] Pihur V., Datta S., Datta S. (2009). RankAggreg, an R package for weighted rank aggregation. BMC Bioinform..

[51] Pihur V., Datta S., Datta S. (2007). Weighted rank aggregation of cluster validation measures: A Monte Carlo cross-entropy approach. Bioinformatics.

[52] Pihur V., Datta S., Datta S. (2008). Finding common genes in multiple cancer types through meta–analysis of microarray experiments: A rank aggregation approach. Genomics.

[53] Menardi G., Torelli N. (2014). Training and assessing classification rules with imbalanced data. Data Min. Knowl. Discov..

[54] Fu G.H., Yi L.Z., Pan J. (2019). LASSO-based false-positive selection for class-imbalanced data in metabolomics. J. Chemom..

[55] Fu G.H., Zhang B.Y., Kou H.D., Yi L.Z. (2017). Stable biomarker screening and classification by subsampling-based sparse regularization coupled with support vector machines in metabolomics. Chemom. Intell. Lab. Syst..

[56] Ma S.S., Zhang B.Y., Chen L., Zhang X.J., Ren D.B., Yi L.Z. (2018). Discrimination of Acori Tatarinowii Rhizoma from two habitats based on GC-MS fingerprinting and LASSO-PLS-DA. J. Cent. South Univ..

[57] Fernández A., García S., Galar M., Prati R.C., Krawczyk B., Herrera F. (2018). Dimensionality Reduction for Imbalanced Learning. Learning from Imbalanced Data Sets.

